# Insights into Antiviral Candidates against Oropouche Virus: A Molecular Dynamics Study

**DOI:** 10.1021/acsphyschemau.5c00042

**Published:** 2025-07-23

**Authors:** Guilherme Colherinhas, Wesley B. Cardoso

**Affiliations:** Instituto de Física, 67824 Universidade Federal de Goiás, 74.690-900 Goiânia, Goiás, Brazil

**Keywords:** molecular dynamics, Oropouche virus, Orthobunyavirus, molecular docking, HIV inhibitors

## Abstract

The Oropouche virus (OROV), an emerging arbovirus from the Peribunyaviridae family, represents a growing public health concern in Latin America, particularly due to its rapid urban spread and lack of specific treatments. In this study, we employed an integrated computational strategy combining molecular docking and molecular dynamics (MD) simulations to evaluate the potential of HIV protease inhibitors as candidates for repurposing against the Gc glycoprotein of OROV, a critical component in viral fusion and host cell entry. While docking initially ranked Saquinavir as the top binder, subsequent MD simulations revealed that nelfinavir and indinavir exhibited superior performance across multiple criteria, including binding energy, structural stability, center-of-mass distance maintenance, and consistent hydrogen bonding. These findings emphasize the limitations of docking-only approaches and highlight the importance of dynamic and energetic analyses for accurate inhibitor selection. The proposed computational pipeline demonstrates its value in identifying stable, high-affinity ligands and offers a promising route for accelerating drug discovery against neglected viral diseases such as OROV.

## Graphical abstract

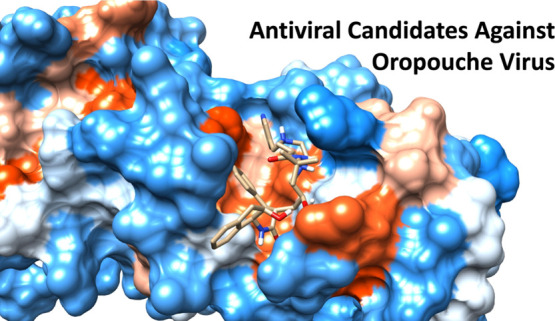

## Introduction

The Oropouche virus (OROV), an arbovirus of the *Orthobunyavirus* genus and the Peribunyaviridae family, is currently recognized as one of the most neglected emerging infectious diseases in Latin America. First identified in 1955, OROV was historically restricted to the Amazon region, but in recent decades has demonstrated increasing epidemic potential, reaching densely populated urban areas and presenting a real risk of global expansion.^1^ Despite this, it lacks robust diagnostic infrastructure, blood bank screening protocols, and the development of specific therapies, being systematically excluded from international surveillance priorities.^2,3^

Increasing human mobility, deforestation, climate change, and the uncontrolled urban spread of vectors such as *Culicoides paraensis* have contributed to the amplification of OROV outbreaks in both number and intensity.^1,4,5^ In 2024 and 2025, more than 25,000 cases were confirmed by RT-PCR across 13 countries, with significant spread in Brazil—including states outside the Amazon—and reports of deaths and fetal malformations associated with the infection.^6–8^ The disease typically presents as an acute febrile syndrome, often accompanied by headache, myalgia, and photophobia, but in more severe cases, it progresses to encephalitis, meningitis, and recurrent neurological symptoms.^8,9^ These overlapping symptoms with other arboviruses such as dengue, Zika, and chikungunya complicate the clinical diagnosis.^4,10^

In response to this neglected emergency, various strategies are being explored to develop antiviral therapies against OROV. A promising example is wedelolactone, a natural compound that showed potent inhibition of the viral RNA polymerase, as well as a concentration-dependent reduction in viral titer in in vitro assays.^11^ Similarly, acridone derivatives FAC21 and FAC22 inhibited viral replication by 99.9%, interfering with endonuclease activity and suggesting dual mechanisms of action, including dsRNA intercalation.^12^ These studies demonstrate the importance of rational drug design and natural product screening as effective antiviral discovery strategies.

In addition, natural venom-derived compounds have emerged as innovative antiviral agents. Enzymes such as phospholipase A_2_ and crotoxin from snake venom have demonstrated direct virucidal activity against OROV, disrupting the viral envelope and preventing host cell entry.^13,14^ Other orthobunyavirus-related protein fragments, such as the C4 fragment of the Schmallenberg virus nucleoprotein, have elicited cross-protective immune responses in mice against OROV, offering perspectives for the development of pan-orthobunyavirus vaccines.^15^

On the epidemiological front, the emergence of the AM0088 lineage, which is more virulent and less effectively neutralized by antibodies, underscores the importance of sustained molecular surveillance and genome monitoring.^7,16^ Although surveillance studies in Colombia did not detect active OROV circulation, its inclusion in differential diagnoses of acute febrile illnesses remains essential.^17^ The increasing frequency of outbreaks in the Amazon basin and Caribbean regions demands coordinated public health policies, improved clinical training, and effective outbreak containment strategies.^9,18^

OROV also represents a potential threat to transfusion safety, particularly in the absence of systematic blood screening for this virus across Latin America.^3^ Limited laboratory capacity in endemic areas, coupled with the lack of standardized commercial diagnostic kits, remains a barrier to effective detection and surveillance.^2^ While diagnostic advances such as biosensor technology have been applied to other emerging viruses like monkeypox,^19^ no equivalent technology is yet available for OROV. The lessons learned from the COVID-19 pandemic have shown that prior investment and diagnostic preparedness are critical to controlling neglected viral outbreaks.^2^

Given these challenges, it is imperative to prioritize the OROV within research, innovation, and public health frameworks. The establishment of dedicated research centers, such as CRAFT in Brazil, which combines structural biology, fragment-based screening, and artificial intelligence for neglected disease drug discovery, is a critical step in addressing viruses like OROV.^20^ To mitigate the growing public health impact of this virus, it is crucial to accelerate vaccine development, expand genomic and clinical surveillance networks, decentralize diagnostic testing capacity, and ensure access to safe and effective therapeutic interventions. Recognizing the urgency of OROV research is not only a scientific necessity but also an ethical and global health imperative.

In this context, computational tools have become essential in the rational design of new antiviral therapies, offering speed, cost efficiency, and predictive power. Molecular docking and molecular dynamics (MD) simulations allow researchers to assess the interaction potential and stability of candidate drugs within key viral targets such as the endonuclease domain of the OROV polymerase. Docking simulations generate initial binding affinity estimates, while MD simulations provide detailed insights into conformational dynamics and thermodynamic properties over time. These techniques have already been applied successfully in the identification of wedelolactone and acridone derivatives with antiviral potential against OROV. Therefore, this project proposes a comprehensive docking and MD analysis of OROV with a panel of antiviral candidates, aiming to evaluate their binding stability and inhibitory potential. By identifying stable and energetically favorable protein-ligand complexes, we prioritized the most promising molecules for further experimental validation. This integrative computational strategy will serve as a robust foundation for accelerating the discovery of antiviral agents targeting neglected arboviruses, such as OROV.

## Methodology

### System Preparation and Molecular Docking

The three-dimensional structure of the OROV glycoprotein Gc head domain was obtained from the RCSB Protein Data Bank (PDB ID: 6H3X).^21^ This structure was resolved using X-ray crystallography at a temperature of 277.15 K,^22^ with a resolution of 2.09 Å, and prepared using vapor diffusion in a hanging drop configuration. The crystallographic model represents a biologically relevant domain of the OROV glycoprotein, which plays a critical role in host–cell interactions and thus serves as a potential therapeutic target. To prepare the protein structure for molecular docking, the PDB file was imported into UCSF Chimera software.^23^ All nonstandard residues, water molecules, and heteroatoms were removed since these elements are not essential for the ligand-binding simulation and may interfere with accurate docking. The protein was then processed using the DockPrep module,^24^ which assigns appropriate charges, adds missing atoms, and optimizes side-chain conformations. The resulting model was saved in the MOL2 format, a file type compatible with ligand-based docking workflows. Ligands used in this study—primarily HIV protease inhibitors repurposed as antiviral candidates—were retrieved from the PubChem database^25^ in either 2D (SDF) or 3D formats. In cases where only 2D representations were available, the ligands were converted into 3D structure files using Open Babel^26^ with the --gen3D option, ensuring correct geometry generation for docking. Each ligand was energy-minimized and prepared for docking by using default parameters. Molecular docking simulations were performed using the AutoDock Vina^27,28^ plugin available within Chimera.^23^ The docking grid box was centered at the coordinates (22, 13, 54) and defined with a box size of 46 × 51 × 69 Å, which encompassed the entire surface of the protein, allowing exhaustive exploration of possible binding pockets. Docking results were evaluated based on root-mean-square deviation (RMSD) values and the number of hydrogen bonds formed. The conformation with the lowest binding energy and most favorable interactions was selected for the MD simulations.

### Topology Generation and System Assembly

To construct the MD system, the CHARMM36-jul2022 all-atom force field^29^ was selected for the protein, in combination with the TIP3P water model^30^ for solvent molecules. The protein topology was generated using the “pdb2gmx” module in GROMACS software,^31^ where hydrogen atoms were omitted initially but were automatically added during solvation. The processed OROV glycoprotein consists of 221 residues and 3580 atoms, with a total mass of approximately 25750 atomic mass units (a.m.u.) and a net charge of −3*e*. The ligand topologies were generated independently using the SwissParam^32,33^ web server, which provides CHARMM-compatible topology and parameter files based on the ligand’s MOL2 input. SwissParam output includes partial atomic charges, atom types, bonded connectivity, and dihedral angles required for integration into the GROMACS topology file. The resulting *.itp and coordinate files were added to the simulation topology (topol.top) and included in the system using a combined structure (complex.gro) that merged protein and ligand coordinates. The simulation box was constructed with dimensions of 6.052 × 6.614 × 8.181 nm, yielding a total volume of 327.47 nm^3^. This box size ensures that the protein-ligand complex remains sufficiently distant (at least 1.0 nm) from the box boundaries, avoiding artifacts from periodic images during MD simulations.

### Solvation and Ion Addition

The simulation box was solvated using the TIP3P water model^30^ by means of the “gmx solvate” command in GROMACS software. A total of 9516 water molecules were added to fill the simulation box, resulting in a system with 32,206 atoms distributed among 9751 residues. The resulting system had a calculated density of 1012.79 g/L, close to that of human plasma. To neutralize the system and mimic physiological ionic strength, sodium and chloride ions were introduced using the “gmx genion” module of GROMACS. The target salt concentration was set to 0.154 mol/L, corresponding to a 0.9% NaCl solution (normal saline), which reflects the ionic environment of human blood. Ion addition was performed with the “-conc 0.154 -neutral” options. The number of water molecules replaced by ions varied slightly, depending on the ligand and the system charge. Figure 1 shows all antiviral compounds used in MD simulations against OROV: (a) lopinavir, (b) nelfinavir, (c) tipranavir, (d) fosamprenavir, (e) indinavir, (f) atazanavir, (g) darunavir, (h) amprenavir, (i) saquinavir, and (j) ritonavir. For instance, in the systems containing amprenavir, atazanavir, darunavir, fosamprenavir, lopinavir, ritonavir, and tipranavir, 63 water molecules were replaced with 33 Na^+^ and 30 Cl^–^ ions. For indinavir, nelfinavir, and saquinavir, replacements were 61–62 solvent molecules, maintaining the same ion ratio.

**Figure 1:**
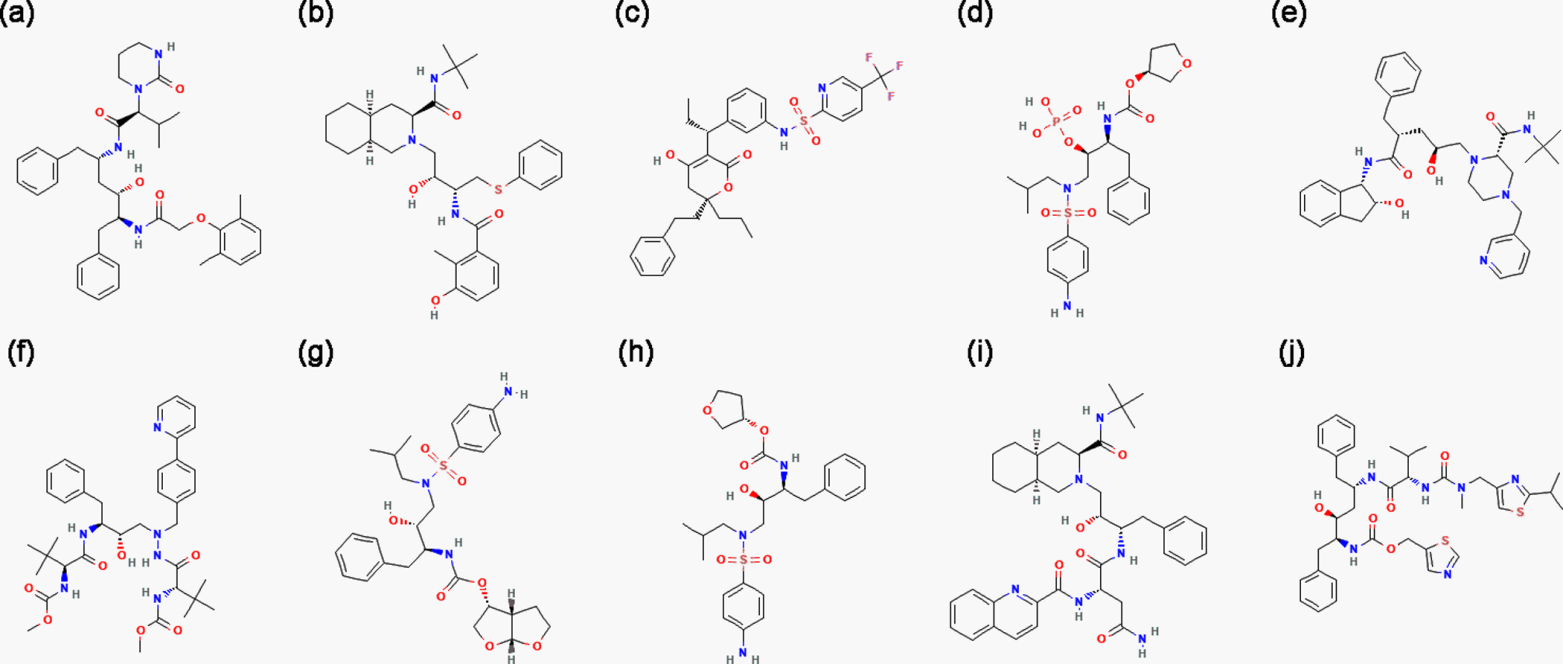
Antiviral compounds used in MD simulations against OROV. The compounds are (A) lopinavir, (B) nelfinavir, (C) tipranavir, (D) fosamprenavir, (E) indinavir, (F) atazanavir, (G) darunavir, (H) amprenavir, (I) saquinavir, and (J) ritonavir.

To ensure reproducibility and provide a comprehensive overview of the simulation systems, we summarize the key parameters of each protein-ligand complex used in the MD simulations in Table 1. The table includes the total number of atoms, initial system density after solvation, number of water molecules, and number of sodium and chloride ions added to neutralize the system. All systems were neutralized to a net charge of zero through the addition of the appropriate counterions. The simulation box dimensions were kept constant across all systems, as described in section Topology Generation and System Assembly.

**Table 1:** Summary of the System Parameters for Each Protein-Ligand Complex Used in MD Simulations^a^

complex	atoms	initial density (g/L)	water molecules	ions (Na+/Cl−)
OROV-amprenavir	3650	1012.11	9523	33/30
OROV-atazanavir	3683	1013.13	9520	33/30
OROV-darunavir	3655	1012.41	9524	33/30
OROV-fosamprenavir	3655	1012.42	9522	33/30
OROV-indinavir	3674	1012.49	9521	31/30
OROV-lopinavir	3674	1012.55	9521	33/30
OROV-nelfinavir	3666	1012.15	9520	32/30
OROV-ritonavir	3678	1012.76	9518	33/30
OROV-saquinavir	3680	1012.60	9519	32/30
OROV-tipranavir	3655	1012.23	9519	33/30

a The total net charge of the system is adjusted to zero through the inclusion of appropriate counterions.

### Energy Minimization and System Equilibration

Following solvation and ionization, energy minimization was performed to eliminate steric clashes and optimize the initial geometry. The minimization was carried out using the steepest descent algorithm until the maximum force dropped below 1000 kJ/mol/nm. The minimized structure served as the starting point for the equilibration and production runs. Equilibration was conducted in two sequential steps to stabilize the temperature and pressure. The first phase employed the NVT ensemble (constant number of particles, volume, and temperature) over a 100 ps simulation at 310 K using a velocity-rescaling^34^ thermostat. This phase allowed the system temperature to equilibrate while keeping the volume fixed. An optional second phase using the NPT ensemble (constant number of particles, pressure, and temperature) could be added to stabilize pressure at 1 atm using the Parrinello–Rahman barostat.^35^

### Production MD Simulations

After successful equilibration, position restraints were removed and production MD simulations were carried out for 50 ns in the NVT ensemble at 310 K. The simulation used a 2 fs time step, particle mesh Ewald (PME)^36^ for long-range electrostatics, and 1.2 nm cutoffs for both van der Waals and Coulomb interactions. Periodic boundary conditions were applied in all directions to emulate a bulk environment. The LINCS algorithm^37^ was employed to constrain all bond lengths involving hydrogen atoms, which allows for an integration time step of 2 fs, ensuring computational efficiency while maintaining the stability of the system during simulation. The constraints imposed by LINCS were applied after each step of the simulation to maintain the geometry of the system, especially for the hydrogen bonds (HBs) that would otherwise be prone to large fluctuations in the bond length. Simulation trajectories were saved every 10 ps and were analyzed using standard GROMACS tools.^31^ Postsimulation analysis included computation of RMSD, HB formation, radius of gyration, interaction energies, and conformational stability of the ligand-protein complex. These data provide insight into the dynamic behavior and binding stability of each ligand, allowing a comparative assessment of antiviral candidates against the OROV glycoprotein.

## Analyses and Results

### Lipinski’s Rule of Five

Lipinski’s rule of five^38^ is a guideline used to distinguish between drug-like and nondrug-like molecules, predicting the likelihood of a compound’s success or failure based on its drug-like properties. The rule suggests that molecules with two or more of the following characteristics have a higher probability of being successful drug candidates: (a) molecular mass less than 500 Da; (b) high lipophilicity (expressed as log *P* less than 5); (c) fewer than 5 hydrogen bond donors; (d) fewer than 10 hydrogen bond acceptors; and (e) molar refractivity between 40 and 130. These parameters are indicative of favorable absorption, distribution, metabolism, and excretion (ADME) properties, which are crucial for the success of a compound as a therapeutic agent. This rule will be applied as an initial screening method for the selection of antiviral candidates in the simulation block involving the Oropouche virus and the described compounds. By employing Lipinski’s rule of five, we aim to filter compounds with favorable drug-like characteristics before conducting more detailed analyses through molecular docking and MD simulations. Alternatively, to evaluate Lipinski’s rule of five for the selected compounds, we utilized the web server available at http://www.scfbio-iitd.res.in/software/drugdesign/lipinski.jsp. The high docked score ligands were used as input for the server, which then generated the Lipinski criterion values for each compound. The results of this analysis, including molecular mass, lipophilicity, hydrogen bond donors and acceptors, and molar refractivity, are summarized in a table, allowing for a quick assessment of their drug-likeness based on the rule of five.

The analysis of the compounds based on Lipinski’s rule of five reveals that several do not fully comply with the established criteria for drug-like properties (Table 2). Most compounds exceed the recommended molecular weight of 500 g/mol, with lopinavir, tipranavir, indinavir, atazanavir, ritonavir, and saquinavir violating this criterion. As for log *P*, compounds such as tipranavir and ritonavir surpass the recommended limit of ≤5. Regarding HB donors, indinavir and saquinavir exceed the limit of 5, while most compounds meet the acceptable range for HB acceptors (≤10). However, fosamprenavir, atazanavir, and ritonavir exceed the 10-acceptor limit. Finally, all compounds show molar refractive values above the recommended range (40–130). Despite these violations, all compounds were simulated with the OROV using MD simulations to assess the potential binding affinity and interactions. This approach aims to evaluate the best possible interaction between the virus and antiviral candidates, despite the deviations from Lipinski’s rule.

**Table 2:** Physicochemical Properties and Molecular Docking Affinities of Selected HIV Protease Inhibitors against the Oropouche Virus Gc Glycoprotein^a^

PubChem CID	name	affinity (kcal/mol)	mol. weight (≤500 g/mol)	log *P* (≤5)	HB donor (≤5)	HB acceptor (≤10)	molar ref. (40–130)
92727	lopinavir	–7.7	627	4.11	4	8	179
64143	nelfinavir	–6.6	568	3.33	5	6	160
54682461	tipranavir	–7.3	602	8.40	2	7	152
131536	fosamprenavir	–7.5	585	3.60	5	12	144
5362440	indinavir	–6.9	615	–0.20	6	7	171
148192	atazanavir	–7.3	703	2.49	5	12	194
213039	darunavir	–6.6	547	3.46	4	10	142
65016	amprenavir	–7.0	505	3.48	4	9	133
441243	saquinavir	–8.5	671	1.68	7	10	187
y392622	ritonavir	–7.1	720	6.11	4	11	198

a The table includes the PubChem CID, compound name, binding affinity (kcal/mol), molecular weight, partition coefficient (log *P*), number of hydrogen bond donors and acceptors, and molar refractivity. The drug-likeness criteria from Lipinski’s rule of five and Veber’s rules are indicated in parentheses for reference.

### Molecular Docking Analysis

The high binding affinity of saquinavir, despite its elevated molecular weight, underscores its potential as a lead candidate. However, its pharmacokinetic properties may require optimization to enhance the bioavailability. The variability in lipophilicity among the compounds highlights the need for structural modifications to achieve a balance between solubility and membrane permeability. The elevated molar refractivity observed in most candidates suggests potential challenges in polarizability, which could be addressed through further chemical refinement.

Table 2 presents the molecular docking results for anti-HIV compounds targeting the Gc domain of the Oropouche virus glycoprotein (PDB: 6H3X). The analysis focused on the binding affinity (kcal/mol) between the compounds and the Gc domain along with their physicochemical properties, including molecular weight, partition coefficient (log *P*), hydrogen bond donors and acceptors, and molar refractivity. The results indicate that saquinavir exhibited the strongest binding affinity for the Oropouche virus (−8.5 kcal/mol), followed by lopinavir (−7.7 kcal/mol) and fosamprenavir (−7.5 kcal/mol). On the other hand, nelfinavir and darunavir showed the lowest binding affinities (−6.6 kcal/mol). These findings suggest that saquinavir may be the most promising compound for inhibiting the glycoprotein Gc, likely due to its strong interactions with the target site.

In terms of physicochemical properties, most of the compounds exceeded the ideal molecular weight of 500 g/mol, with amprenavir (505 g/mol) being the closest to this threshold. The lipophilicity, as reflected by the log *P* values, varied considerably across the compounds. Tipranavir had the highest log *P* (8.40), indicating significant lipophilicity, while indinavir had the lowest value (−0.20), suggesting high hydrophilicity. All compounds complied with the acceptable limits for hydrogen bond donors (≤5) and acceptors (≤10), which are essential for favorable drug-like characteristics. However, most compounds had molar refractivity values above the ideal upper limit of 130, with amprenavir and fosamprenavir being exceptions, as they were closer to the optimal range.

Despite its high molecular weight, saquinavir’s strong binding affinity suggests that it could be a lead candidate for further development. However, its pharmacokinetic properties might need to be optimized for improved bioavailability. The variability in lipophilicity among the compounds suggests that structural modifications may be necessary to balance the solubility and membrane permeability. Additionally, the elevated molar refractivity observed in most compounds could pose challenges related to polarizability, which could potentially be addressed through further chemical refinement.

### MD Analysis

First, to verify that all systems reached thermodynamic equilibrium, we monitored the time evolution of total potential energy, temperature, pressure, and density throughout the 50 ns simulations. These properties are presented in Figure 2. All systems exhibited stabilization of the monitored parameters after an initial equilibration phase with only minor fluctuations around average values. These results confirm that equilibrium was achieved and that the systems remained stable during the production phase of the simulations.

**Figure 2:**
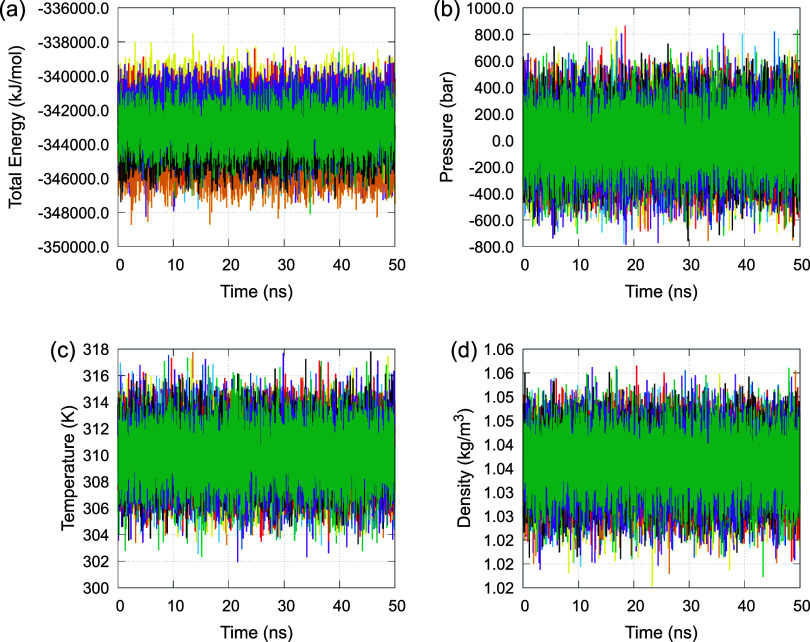
Time evolution of key thermodynamic properties monitored during the 50 ns MD simulations for all protein-ligand complexes: (a) total potential energy; (b) temperature; (c) pressure; and (d) density. Each line represents a different complex: amprenavir (magenta), atazanavir (green), darunavir (light blue), fosamprenavir (orange), indinavir (yellow), lopinavir (dark blue), nelfinavir (red), ritonavir (black), saquinavir (dark magenta), and tipranavir (dark green). All systems reached thermodynamic equilibrium, as indicated by the stabilization of the monitored parameters over time.

Figure 3 illustrates the interaction energies between the OROV glycoprotein Gc and various ligands, as obtained from MD simulations. Specifically, in Figure 3a, we display the Lennard-Jones (LJ) short-range energies, which reflect the van der Waals interactions between the ligands and the glycoprotein. Ritonavir (−157.5 kJ/mol) and tipranavir (−150.8 kJ/mol) exhibited the most favorable LJ energies, indicating strong van der Waals interactions. Their fluctuations were relatively low (17.4 and 9.2% variance, respectively), suggesting stable binding. In contrast, indinavir (−45.3 kJ/mol) and nelfinavir (−102.8 kJ/mol) showed less favorable LJ energies with moderate fluctuations (37.0 and 16.1% variance, respectively).

**Figure 3:**
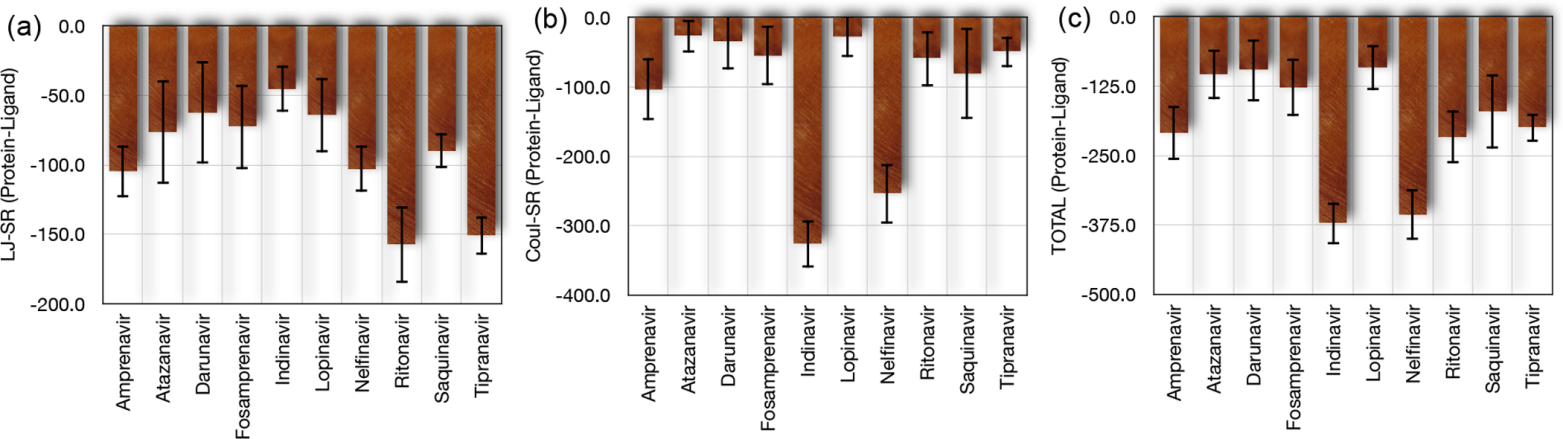
(a) Lennard-Jones, (b) Coulomb SR, and total energies considering the interaction between the Oropouche virus glycoprotein and the different ligands.

Figure 3b presents the Coulombic short-range (Coul-SR) energies, which represent the electrostatic interactions. In contrast with the LJ results, indinavir (−326.5 kJ/mol) and nelfinavir (−254.0 kJ/mol) displayed the most favorable Coul-SR energies, indicating strong electrostatic binding. Their fluctuations were minimal (10.3 and 16.8% variance, respectively), reflecting highly stable interactions. On the other hand, saquinavir (−80.8 kJ/mol) and fosamprenavir (−54.9 kJ/mol) exhibited less favorable Coul-SR energies, with higher fluctuations (81.6 and 77.7% variance, respectively), suggesting less stable electrostatic interactions.

A joint comparison can be made by taking into account the total energy of the above contributions. To this end, Figure 3c combines the LJ and Coulombic energies to show the total interaction energies. Indinavir (−371.8 kJ/mol) and nelfinavir (−356.9 kJ/mol) displayed the most favorable total energies, with minimal fluctuations (10.1 and 12.8% variance, respectively), reinforcing their strong and stable binding affinity. Ritonavir (−216.4 kJ/mol) and tipranavir (−200.1 kJ/mol) also exhibited favorable total energies, with moderate fluctuations (22.3 and 12.9% variance, respectively), primarily driven by their robust LJ interactions. In contrast, lopinavir (−91.7 kJ/mol), atazanavir (−103.3 kJ/mol), and darunavir (−96.7 kJ/mol) showed less favorable total energies and significant fluctuations (43.4, 43.0, and 56.9% variance, respectively), indicating weaker and less stable overall binding.

A comprehensive evaluation of the interaction energies reveals that indinavir and nelfinavir emerge as the most promising candidates, owing to the synergistic contribution of strong electrostatic (Coulombic) and van der Waals (LJ) interactions. These compounds not only achieved the lowest total interaction energies but also maintained minimal energetic fluctuations throughout the simulations, indicating highly stable and sustained binding to the Gc domain of the OROV glycoprotein. In addition, ritonavir and tipranavir displayed favorable binding profiles primarily driven by robust van der Waals interactions coupled with moderate energy stability.

The MD simulations provided detailed insights into the stability, compactness, and binding dynamics of the Oropouche virus in complex with various ligands, as illustrated in Figure 4. Each panel of the figure highlights a specific aspect of the system’s behavior, supported by the quantitative data presented in the table.

**Figure 4:**
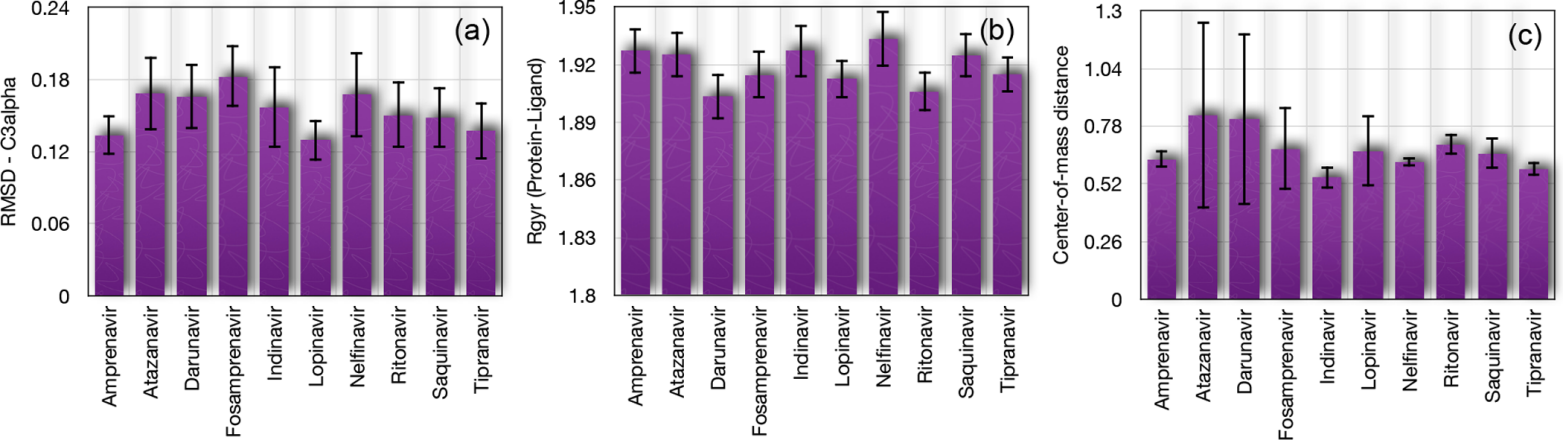
(a) Root-mean-square deviation (RMSD) of C3-α protein atoms. (b) Radius of gyration of the entire system and (c) distance between the center of mass of the ligand atoms with respect to the protease atoms.

Figure 4a shows the RMSD of the C3-α protein atoms, which reflects the stability of the protein backbone during the simulation. Lower RMSD values indicate greater stability, while higher values suggest conformational changes or flexibility. Among the ligands, amprenavir and lopinavir exhibited the lowest RMSD values (0.1335 and 0.1296 Å, respectively), indicating that these ligands maintained the protein structure in a highly stable conformation. In contrast, fosamprenavir showed the highest RMSD (0.1826 Å), suggesting greater flexibility or conformational changes in the protein backbone. The fluctuations in RMSD were relatively small for all ligands, further confirming their stabilizing effect on the protein.

The radius of gyration (Rgyr) of the entire system is presented in Figure 4b, which measures the compactness of the protein-ligand complex. Lower Rgyr values indicate a more compact structure, while higher values suggest expansion or unfolding. The Rgyr values for all systems remained relatively consistent, ranging from 1.9034 Å (darunavir) to 1.9335 Å (nelfinavir). This indicates that the overall compactness of the protein-ligand complexes was maintained throughout the simulations. Darunavir and ritonavir showed the lowest Rgyr values (1.9034 and 1.9060 Å, respectively), suggesting a slightly more compact structure compared to other ligands. The small fluctuations in Rgyr further confirm the stability of the system, with all values below 0.0144 Å.

Subsequently, to investigate the spatial positioning of the ligand atoms relative to those of the Oropouche virus glycoprotein, we analyzed the distance between the centers of mass of the ligand and the Oropouche virus glycoprotein Gc, providing insights into the relative positioning and binding stability of the ligands. The results of this analysis are presented in Figure 4c. Note that a smaller and more stable distance indicates a stronger and more consistent binding interaction. Indeed, tipranavir exhibited the smallest CM distance (0.586 Å) and the lowest fluctuations (5.2%), confirming its highly stable positioning relative to the glycoprotein. Nelfinavir also demonstrated a small CM distance (0.6193 Å) with minimal fluctuations (3.3%), indicating a strong and stable interaction. In contrast, atazanavir and darunavir showed larger CM distances (∼0.83 and ∼0.81 Å, respectively) and significant fluctuations (50.7 and 47.8%, respectively), suggesting weaker or less stable binding. Indinavir and ritonavir displayed moderate CM distances (∼0.55 and ∼0.70 Å, respectively) with low fluctuations (8.8 and 6.9%, respectively), indicating stable but slightly dynamic positioning. We highlight that the percentage fluctuation of the receptor-ligand complex provides an additional insight into the stability of the interaction.

An evaluation of the interaction energies reveals that indinavir and nelfinavir stand out as promising candidates due to the synergistic contribution of electrostatic (Coulombic) and van der Waals (LJ) interactions. These compounds not only exhibited the most favorable total interaction energies but also maintained minimal energetic fluctuations throughout the simulations, indicating highly stable and sustained binding to the Gc domain of the OROV glycoprotein. Additionally, ritonavir and tipranavir demonstrated favorable binding profiles, primarily driven by van der Waals interactions combined with a moderate energy stability. Furthermore, indinavir, nelfinavir, tipranavir, and ritonavir not only excelled in terms of interaction energy but also preserved favorable structural characteristics such as low RMSD fluctuations, compact protein conformations (as indicated by the radius of gyration), and small, stable center-of-mass distances. Notably, tipranavir and nelfinavir exhibited the most stable spatial positioning relative to the glycoprotein with minimal fluctuations, reinforcing their potential as strong and persistent binders. Altogether, the energetic and structural analyses converge to identify these compounds—especially indinavir, nelfinavir, tipranavir, and ritonavir—as leading candidates for future investigations in the context of OROV inhibition.

Figure 5 provides a comprehensive view of the interaction dynamics between the Oropouche virus glycoprotein Gc and the ligand atazanavir. Figure 5a illustrates the temporal evolution of the center-of-mass (COM) distance, revealing an initial sharp increase in separation followed by a stabilization at a lower distance. The red-highlighted region marks the time frame where the most significant variation in COM distance occurs, indicating a possible unbinding or structural rearrangement event. Figure 5b,c offers molecular representations corresponding to two distinct points in the trajectory, at approximately 9 and 40 ns, respectively. In Figure 5b, the ligand appears spatially separated from the glycoprotein, consistent with the peak in COM distance observed in Figure 5a. In contrast, Figure 5c depicts a configuration where the ligand is close to the glycoprotein, aligning with the lower COM distance values in the latter part of the trajectory. This suggests that after an initial displacement, the ligand undergoes a reassociation or stabilizing interaction with the glycoprotein.

**Figure 5:**
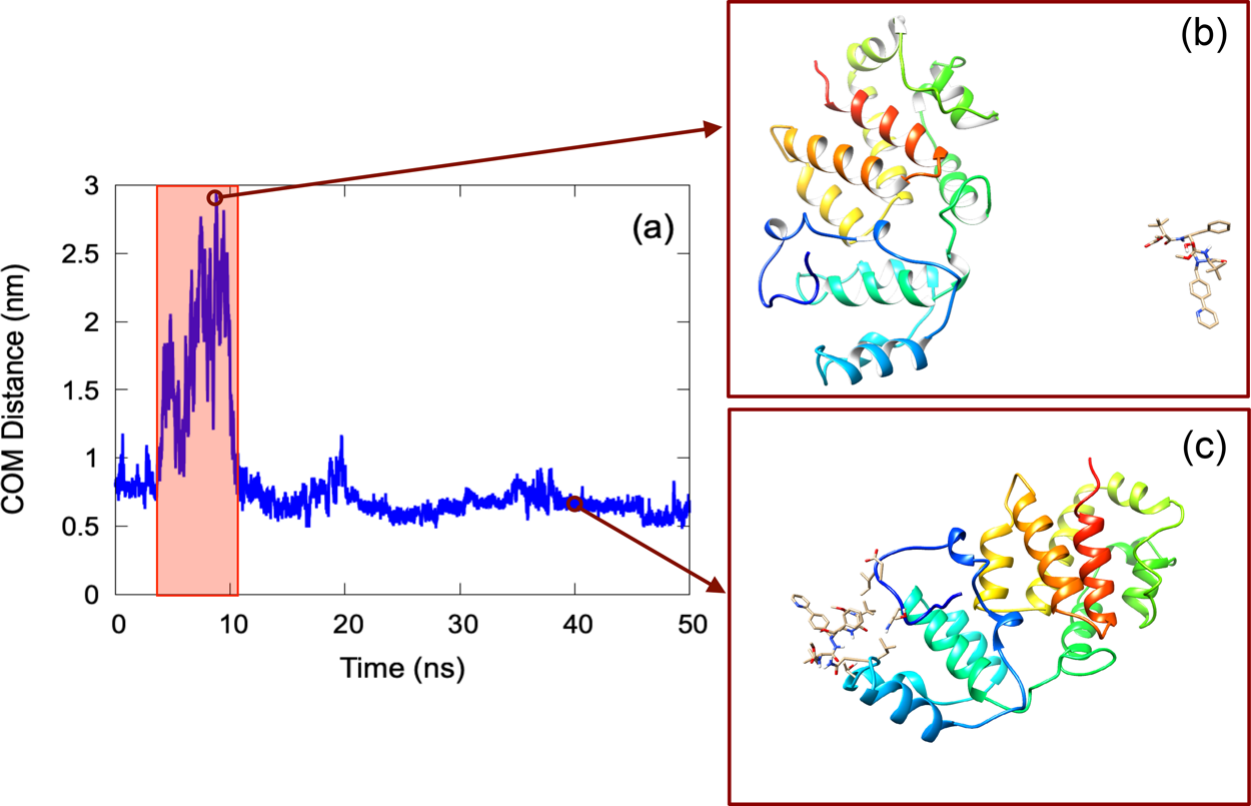
(a) Temporal evolution of the center-of-mass (COM) distance between the Oropouche virus glycoprotein Gc and the ligand (Atazanavir). A highlighted red region emphasizes the interval where the most significant increase in the COM distance is observed. (b) and (c) Two distinct molecular configurations (at 9 and 40 ns, respectively) illustrating the spatial positioning of the ligand in the vicinity of the Oropouche virus glycoprotein Gc for different COM distance values.

The root-mean-square fluctuation (RMSF) profiles derived from MD simulations of the Oropouche virus glycoprotein Gc head domain complexed with various HIV protease inhibitors displayed in Figure 6 reveal differential residue flexibility patterns that reflect ligand-specific modulation of protein dynamics. Notably, atazanavir exhibited higher fluctuations at the N-terminal residues (up to ∼0.48 nm at residue 1), indicating greater local flexibility, while indinavir and nelfinavir showed moderately reduced terminal mobility with maximum RMSF values near 0.3 nm. Across all complexes, core residues within the binding region maintained relatively low RMSF values (mostly below 0.15 nm), suggesting effective stabilization by the inhibitors.

**Figure 6:**
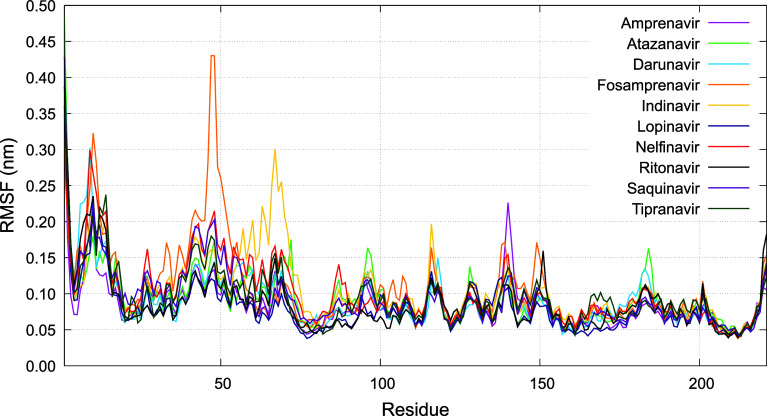
Per-residue root-mean-square fluctuation (RMSF) profiles of the Gc glycoprotein for each protein-ligand complex. RMSF values reflect the flexibility of each residue’s *C*_α_ atom. Higher fluctuations are observed mainly at terminal and loop regions, while residues in the core and binding sites exhibit low mobility, indicating stable protein–ligand interactions.

Distinct peaks of increased flexibility were observed in loop regions around residues 40–70 for all ligands, with fosamprenavir showing slightly higher fluctuations (∼0.43 nm at residue 47) compared to others. In this region, indinavir presented a higher RMSF (residue 67), with a value of around 0.3 nm. Nelfinavir, on the other hand, had its highest value for RMSF in residue 9. These RMSF patterns suggest that while all inhibitors stabilize key structural elements, they differ in modulating peripheral dynamics, which could influence their binding affinity and antiviral efficacy.

The HB formation between the OROV glycoprotein Gc and potential inhibitor candidates was quantitatively evaluated with results presented in Figure 7. Indeed, the HB analysis provides crucial insights into the molecular basis of ligand–protein interactions, complementing the structural and energetic evaluations. Nelfinavir emerged as the most effective HB former, exhibiting the highest average number of HBs (3.4 ± 1.1) with moderate fluctuation (32.2%). This suggests a stable and robust HB network between the ligand and the glycoprotein. Also, indinavir also demonstrated strong HB capability (2.22 ± 0.45 bonds) with remarkably low fluctuation (20.2%), indicating particularly stable interactions. Amprenavir showed intermediate HB formation (2.01 ± 0.92 bonds), though with higher variability (45.6%). Ritonavir and saquinavir displayed similar HBs counts (1.4 ± 1.1 and 1.3 ± 1.0 bonds, respectively) but with substantial fluctuations (75.4 and 82.5% variability), suggesting less consistent HBs maintenance during MD simulations. The remaining compounds—atazanavir, darunavir, lopinavir, and tipranavir—showed limited HBs formations (<1 bond on average) with particularly high fluctuations (>90% variability). Lopinavir exhibited the poorest performance in this regard (0.51 ± 0.79 bonds, 154.4% fluctuation), indicating very weak and unstable HBs interactions. These HBs characteristics correlate well with previous analyses of binding energies and structural stability. Notably, nelfinavir and indinavir, which showed strong hydrogen bonding, also demonstrated favorable binding energies and stable complex formation in earlier analyses. Conversely, compounds with poor hydrogen bond formation had a tendency to show weaker overall binding characteristics. The combination of these analyses suggests that nelfinavir and indinavir are particularly promising candidates due to their ability to form multiple stable hydrogen bonds with the glycoprotein target.

**Figure 7:**
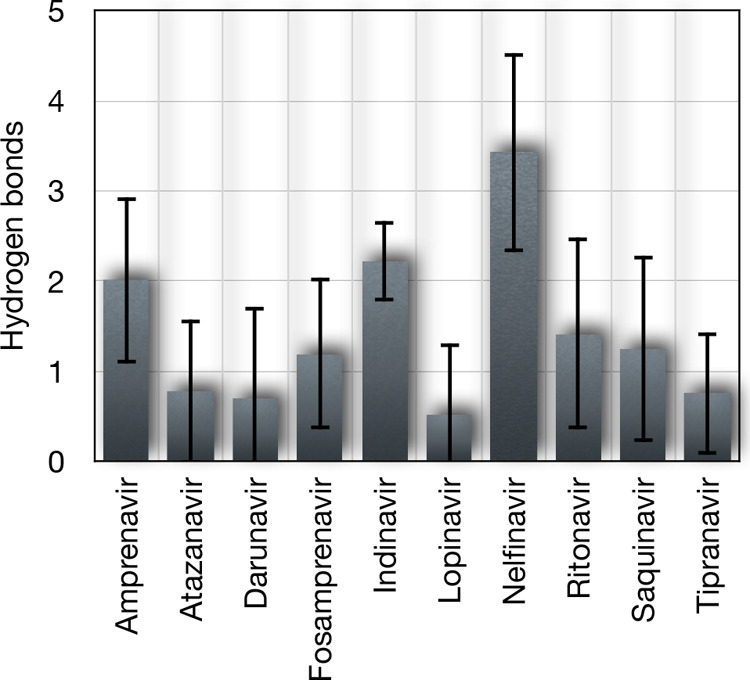
Average number of HBs formed between the glycoprotein Gc of the Oropouche virus and potential inhibitor candidates. Error bars represent the RMSD of HB fluctuations during MD simulations (50 ns).

Building upon the previous energetic and structural analyses, the HB evaluation further reinforces the identification of nelfinavir and indinavir as particularly promising inhibitors of the OROV glycoprotein Gc. Nelfinavir demonstrated the highest average number of hydrogen bonds with moderate fluctuation, indicating a well-established and relatively stable HB network throughout the simulations. This finding is especially significant when considered alongside nelfinavir’s strong electrostatic and van der Waals interactions, minimal COM fluctuations, and compact protein-ligand complex geometry. Similarly, indinavir exhibited a stable and consistent HB profile with one of the lowest fluctuation rates among the tested compounds. The ability of these ligands to form multiple stable hydrogen bonds provides a critical molecular basis for their sustained binding affinity and structural stabilization of the protein-ligand complex. These results highlight that the high interaction energies observed are not solely due to nonbonded interactions but are also supported by directional and persistent HBs that help anchor the ligand within the binding pocket.

To further assess the persistence and stability of ligand–protein interactions during the MD simulations, we computed the time evolution of the number of HBs formed between each ligand and the Gc glycoprotein over 50 ns trajectories. The results are presented in Figure 8, highlighting distinct interaction profiles for the ten studied inhibitors. Notably, nelfinavir and indinavir maintained a relatively high and stable number of Hbs throughout the simulations, suggesting stronger and more persistent interactions with the target protein. These findings complement the binding energy and RMSF analyses, providing additional support for the potential of these compounds as effective inhibitors.

**Figure 8:**
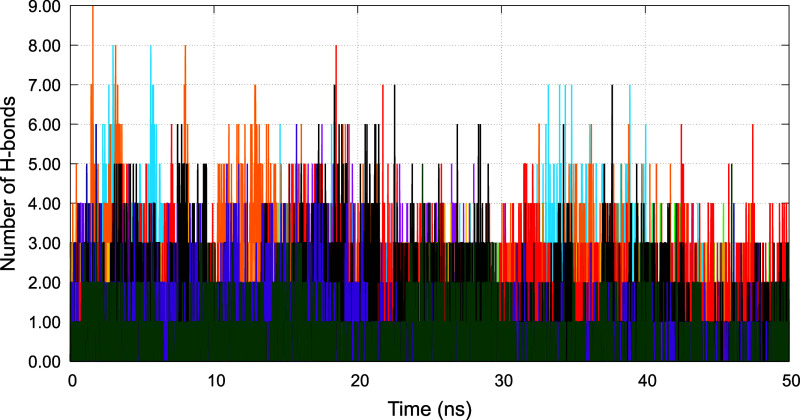
Time evolution of the number of HBs between the Gc head domain of the Oropouche virus and each HIV protease inhibitor during the 50 ns MD simulations. Each line represents a different complex: amprenavir (magenta), atazanavir (green), darunavir (light blue), fosamprenavir (orange), indinavir (yellow), lopinavir (dark blue), nelfinavir (red), ritonavir (black), saquinavir (dark magenta), and tipranavir (dark green).

## Conclusions

This investigation employed integrated molecular docking and MD simulations to evaluate HIV protease inhibitors as potential inhibitors of the Oropouche virus glycoprotein Gc. The results revealed critical discrepancies between the initial docking predictions and observed dynamic behaviors. While molecular docking identified saquinavir as the top-ranked candidate (−8.5 kcal/mol binding affinity), more physiologically reliable MD simulations demonstrated nelfinavir and indinavir to be superior candidates. Nelfinavir emerged as the most stable compound, exhibiting strong binding energy (−356.9 kJ/mol), minimal COM distance fluctuation (3.3%), and persistent HBs (3.43 bonds). Indinavir, despite modest docking performance, showed exceptional structural stability (protein backbone RMSD 0.1573 Å, low COM fluctuation (8.8%), and favorable binding energy (−371.8 kJ/mol)). Comparative analysis revealed that docking-optimized compounds such as atazanavir, darunavir, fosamprenavir, and lopinavir displayed substantial COM distance fluctuations (>23%) and HB variability (>70%), correlating with ligand dissociation events during simulations. These findings establish that viable inhibitors must simultaneously satisfy three key criteria: (1) strong binding energies, (2) COM fluctuations <10%, and (3) consistent HB patterns. The study demonstrates that nelfinavir and indinavir uniquely meet all stability requirements, identifying them as prime candidates for experimental validation against the OROV infection. This integrated computational approach provides a robust screening protocol where MD serves as an essential validation step, effectively eliminating docking false positives and identifying compounds with genuine therapeutic potential through a comprehensive evaluation of binding stability under dynamic conditions.

Furthermore, this study highlights the importance of a multidimensional assessment framework that goes beyond docking scores to capture the complex and dynamic nature of ligand–protein interactions. By integration of analyses of interaction energies, protein conformational stability (RMSD, Rgyr), spatial positioning (COM distances), and HB behavior, the investigation provided a holistic view of binding efficacy. The convergence of these independent yet complementary metrics consistently pointed to nelfinavir and indinavir as the most robust binders. Notably, these compounds not only exhibited favorable interaction profiles in isolation but also maintained structural compactness and persistent, low fluctuation contact with the glycoprotein target, suggesting a strong potential to resist dissociation and maintain inhibitory function under physiological conditions.

These findings offer valuable guidance for the rational design and experimental screening of antivirals targeting OROV and potentially other bunyaviruses. The methodological framework employed here—particularly the use of MD to filter out docking artifacts—can be generalized to future in silico drug repurposing pipelines. As nelfinavir and indinavir are already approved for clinical use, their repurposing could significantly accelerate the development of effective therapeutic strategies against emerging arboviral threats such as OROV. Experimental validation through in vitro binding assays, viral replication inhibition studies, and subsequent in vivo models will be the next essential steps to confirm their antiviral efficacy and therapeutic viability.
